# On the Unfairness of the ‘Fair-share Principle’ for Health Research

**DOI:** 10.1093/phe/phag004

**Published:** 2026-03-26

**Authors:** Victoria Min-Yi Wang

**Affiliations:** University of Edinburgh

## Abstract

How ought scarce health research resources be allocated, where health research spans basic, translational, clinical, health systems and public health research? In this article, I first outline a previously suggested answer to this question: the ‘fair-share principle’ stipulates that total health research funding ought to be allocated in direct proportion with suffering caused by each disease. Second, I highlight a variety of problems the fair-share principle faces. Like other resource allocation frameworks, the principle needs to address the aggregation and distribution of harms and consider cost-effectiveness. Moreover, to make resource allocation recommendations, the principle has to be used in conjunction with real-world estimates of ‘suffering’, usually provided by the Global Burden of Disease Study. These estimates are disease-centric and only take ‘proximal’ causes of health loss into account. Applying the principle based on such estimates disregards ‘distal’ causes, including social determinants of health, thus skewing resource allocation towards biomedical research and away from public health research. Since public health research aims at improving population health while reducing health inequalities, the principle leads to inequitable priority-setting. The fair-share principle can only become equitable when due consideration is also given to ‘distal’ causes that are amenable to public health research and interventions.

## Introduction

The questions of how societies ought to prioritise health *research* and how to allocate and ration scarce health research resources are urgent and receive relatively little attention. Health research is here understood broadly, following the World Health Organization (WHO), which defines health research as ‘the development of knowledge with the aim of understanding health challenges and mounting an improved response to them’ (2012: 5). This development of knowledge spans basic, translational, clinical, health systems and public health research (for this taxonomy, see Pratt and Hyder, 2017). Accordingly, the health research resources in question include funding for different research programmes and research areas, scientists and technicians, computing power, laboratory space, research animals, clinical trial resources, experimental public health intervention resources etc.^1^

Calls from within academia and non-governmental organisations to provide ethical analyses of health research priority-setting (e.g. Pratt and Hyder, 2017; Pratt *et al.*, 2018; World Health Organization, 2025) as well as global health and political events are beginning to raise the profile of these questions. For example, discussions concerning health research prioritisation came to the fore during the COVID-19 pandemic. Various funding bodies, including the US and UK governments, provided extra funding for laboratory-based biomedical/translational research and clinical research on therapies and vaccines, awarding these grants at extraordinary speed (Chinnery *et al.*, 2021). This re-prioritisation and the ‘pivoting’ of researchers from their areas of expertise to COVID-19 research was seen by some as ‘covidization’, risking loss of expertise to study other infectious diseases (Pai, 2020; Prudêncio and Costa, 2020) and non-communicable diseases, in part due to loss of funding (BBC News, 2020).^2^ As a public health emergency, the pandemic also boosted investment into research in epidemiology, the social sciences (e.g. on mental health impacts), and public health (e.g. on public health communications) (Bucher *et al.*, 2023). Due to its global impact and the urgency to act in response, the pandemic highlighted the complexity of health research priority-setting. However, priority-setting is an ongoing and constitutive feature of health policy. In policy circles, debates about health research resource allocation, and health spending more generally, often surface a tension between public health priorities and individual-level curative approaches (e.g. Schmidt, Gostin and Emanuel, 2015).^3^ And yet, with a few exceptions, philosophers have largely not contributed to giving a substantive answer to the pressing question of how health research priorities ought to be set.

It is perhaps unsurprising that an infectious disease pandemic led to an uptick in research funding for epidemiological and public health research related to COVID-19. But as we know from decades of health research, most adverse health outcomes, including non-communicable diseases, fall to some extent within the remit of public health. Since the mid-twentieth century, different theoretical approaches to epidemiology have carved up the landscape of disease causation in a number of ways—ranging from the earlier ‘web’ of disease causation to later social epidemiological theories (cf. Krieger, 2024). No matter the precise approach favoured, however, the frameworks all agree that disease causation is complex; adverse health outcomes are never the result of a single cause.

In parallel with these developments in epidemiological and public health theory, there has been much philosophical debate about how to measure disease burden and health outcomes (e.g. Lenard and Straehle, 2012; Eyal *et al.*, 2013, 2020b). For example, there have been extensive discussions of the development of the disability-adjusted life year (DALY) as an index of health loss that takes both morbidity and mortality into account. Some of these debates concern the various ethical values embedded within the DALY and how they affect the use of the index in health policy (e.g. Voigt, 2012; Schroeder, 2017; Solberg *et al.*, 2020).

In what follows I bring these debates, which have often proceeded in isolation, into conversation with one another by considering health research priority-setting through the lens of the fair-share principle. First, I describe the fair-share principle, which stipulates that total health research funding ought to be allocated in direct proportion with suffering caused by each disease. I then highlight a variety of problems the principle faces. I divide these problems into ‘internal’ and ‘external’ problems. Internal problems refer to those problems that beleaguer most, if not all, resource allocation frameworks, including problems of aggregation and distribution of harms, and considerations of cost-effectiveness. External problems, on the other hand, refer to those that arise when users of the principle start drawing on real-world estimates of budget sizes and disease burden to make judgements about current funding priorities or to recommend alternative resource distributions. I illustrate these external problems by discussing the architecture of a frequently used source of disease burden estimates: the Global Burden of Disease (GBD) Study (Tichenor and Sridhar, 2020; Eyal *et al.*, 2020a). I show that the way in which causes of health loss are represented in epidemiological studies like the GBD is value-laden, and that this value-ladenness affects the appropriateness of using these studies for health policy, and for health research priority-setting in particular. This hitherto overlooked point is comparable to discussions of the value-ladenness and appropriateness of using the DALY metric for health policy.

My argument hinges on the premise that these disease burden estimates are disease-centric and privilege ‘proximal’ causes of health loss. As such, they tend to disregard population and public health research that has long stressed the role of the social determinants of health. Therefore, in drawing on these estimates, the fair-share principle not only risks overlooking important lessons coming from population and public health research but also ultimately fails to meet the standards of fairness in the allocation of funding it was striving to achieve. Building on these critiques, I conclude that the fair-share principle, in conjunction with currently available epidemiological studies like the GBD, is not an ideal to aim for when setting the health research agenda. Instead, the principle might be a helpful tool for guiding deliberation about priority-setting when the disease burden estimates on which it draws are suitably enlarged to include ‘distal’ and social causes of health loss.

## The Fair-share Principle for Health Research

Together with collaborators, the philosopher Philip Kitcher has advanced the fair-share principle as a regulative principle to guide decision-making about the distribution of finite resources (i.e. funding) to health research. The principle stipulates, roughly, that health research funding ought to be allocated in direct proportion with suffering caused by each disease. Kitcher’s main concern in articulating this principle is the mismatch that has been characterised as the 10/90 gap in health research—‘less than 10 per cent of this [global spending on health research] is devoted to diseases or conditions that account for 90 per cent of the global disease burden’ (Global Forum for Health Research, 2000: 1)—which continues to persist (Adam *et al.*, 2023). Other philosophers have also pointed out the ‘disparities’ (Resnik, 2004) and ‘distortions’ (De Winter, 2012) of the health research landscape. And many other researchers—in global and public health, bioethics, health systems analysis, health policy and the health sciences (e.g. Murray, 1994; Stuckler *et al.*, 2008; Gillum *et al.*, 2011; Evans, Shim and Ioannidis, 2014; Kinge *et al.*, 2014; Hanna, 2015; Yao *et al.*, 2015; Ballreich *et al.*, 2021; Millum, 2023; Oliveira *et al.*, 2023; Pandey and Adhikari, 2023; UK Clinical Research Collaboration, 2023; Madsen and Andersen, 2024; Schmallenbach *et al.*, 2025)—discuss or invoke the fair-share principle, but I will rely on Kitcher’s definition because it is a clear statement of the view (Kitcher, 2004, 2011; Reiss and Kitcher, 2009).

I share Kitcher’s concern about the 10/90 gap. In what follows, I am not suggesting that the status quo is better than what the fair-share principle aims to achieve.^4^ Indeed, I will conclude that using the principle as a starting point for deliberation would probably make health research funding distributions more equitable, but only if the values that are baked into the representation of causes of health loss are expanded in the way I discuss below.

In its most recent iteration, the fair-share principle states that:

Waiving considerations of tractability, each disease should be investigated according to its contribution to the total suffering caused by disease. … However the contributions [to suffering] are assessed, if the principle is applied directly to the statistics on disease incidence, it is evident that actual research into diseases is skewed toward conditions affecting affluent people. Many diseases that kill or incapacitate poor people receive support on the order of one-hundredth of their fair share. (Kitcher, 2011: 122)

How should this principle be interpreted? In particular, how should ‘total suffering’ be understood, measured and operationalised? Flory and Kitcher ‘measure the alignment between disease burden and the directions of biomedical research’ (2004: 39) by comparing the share of the total global budget for health research that goes towards researching malaria and tuberculosis with the share of the total number of deaths attributed to these diseases. From this, Flory and Kitcher conclude that malaria and tuberculosis are not receiving their fair share of research investment. (Notably, the size of the budget is extracted from a report, which explicitly states that this estimate includes funding for the ‘medical and natural sciences as well as social sciences including economics and behavioural science’ (Global Forum for Health Research, 2001: 3). A central aim of this report was to create a classification system for health research funding that would go beyond biomedical research and include health systems research and research on ‘health determinants’.)

Since many people die of these diseases prematurely, and both those who do and don’t die suffer from the diseases in other ways, Flory and Kitcher also use the DALY as a proxy for ‘total suffering’. For a given condition, DALYs are calculated by adding the years of life lost to premature mortality (YLLs), calculated against a reference life expectancy, to the years of life lived with disability (YLDs). The larger the DALY, the larger the health loss. Based on DALY estimates gathered for the 2000 GBD study (World Health Organization, 2000), Flory and Kitcher conclude that for malaria and tuberculosis, using the total number of deaths or DALYs yields comparable results. This is unsurprising given that tuberculosis and malaria kill large numbers of young people; the YLLs thus contribute a larger share to the DALY than the YLDs.^5^

The authors also make the further point that individual biomedical researchers have a responsibility to address the 10/90 gap by, for example, switching their research focus from arthritis to infectious disease research (Flory and Kitcher, 2004: 60–61). Moreover, they suggest that ‘the biomedical research community in the affluent world has the obligation to modify the current research agenda so as to give much greater weight to investigations into the diseases that produce extraordinary suffering among the poor’ (Flory and Kitcher, 2004: 56).

The fair-share principle thus establishes a direct and proportional link between disease burden and health research investment. Various other authors also assume that estimating disease burden should guide or even determine research priority-setting, often implicitly and sometimes explicitly stating that funding ought to be distributed in direct proportion with disease burden. For example, work by Stuckler *et al*. found that the WHO budget 1994–2007 was ‘heavily skewed toward infectious diseases’ (2008: 1563), awarding 87 per cent of its total budget to infectious diseases despite the fact that they accounted for less than 15 per cent of global mortality and DALY burden. The authors conclude that the WHO’s budget was misaligned with the disease burden. Similarly, in their analysis of National Institutes of Health (NIH) funding, Gillum *et al*. conclude that ‘NIH funding is not better aligned with US disease burden [as measured in DALYs]’ (2011: e16840) in 2006 than in 1999. Finally, Yao *et al.*, in designing an index for assessing this ‘misalignment’ between health research funding and disease burden, assume ‘that to achieve maximal societal benefit, [research] resources should be allocated across the full distribution of illness proportional to the costs those illnesses impose on society’ (2015: 809), where costs are operationalised in terms of DALYs. These examples illustrate that versions of the fair-share principle abound in the literature.^6^ Note that in all these cases, to get the fair-share principle off the ground, its users rely on real-world health research budgets and estimates of ‘total suffering’ (e.g. death rates or DALYs) that have been *attributed* to particular diseases or causes. We will return to this important point below.

## Internal Problems for the Fair-share Principle

Having explicated the fair-share principle, I now turn to some of the problems it faces. Following the terminology proposed in the introduction, I start, briefly, with the internal problems, i.e. those problems that arise for most, if not all, resource allocation frameworks.

### Aggregation and Distribution

Scholars and policy-makers have argued that resource allocation should take into account not only the amount of benefit a given research programme could contribute (i.e. how much suffering could be reduced); priority-setting should also consider the fair distribution of benefits among intended recipients as well as research programmes’ cost-effectiveness (cf. Ottersen and Norheim, 2020). Focussing first on the ethical issue concerning the distribution of benefits: maximising health at the aggregate level is not the only thing we (should) care about. This is reflected in actual people’s preferences to trade off maximal health gains against prioritising those with more severe symptoms/diseases. At least in the case of health *care* resource allocation, empirical studies suggest that people care about the equitable distribution of health benefits (e.g. Shah, 2009; Nord and Johansen, 2014; Gu *et al.*, 2015). It is not unreasonable to suppose that similar preferences apply to the allocation of health *research* resources. People might prefer investing in research for a relatively rare but severe disease rather than for a very mild but widespread adverse health outcome, even if the aggregate benefit of addressing the common condition would have been larger in terms of DALY reduction.

Since the fair-share principle relies on the aggregation of total suffering caused by each disease, it fails to address questions of distributional fairness when it comes to the disease burden experienced by individuals. For example, it turns out that in 2019—by the reckoning of the GBD Study—back and neck pain jointly contributed 3.37 per cent, while malaria and tuberculosis jointly contributed 3.8 per cent of global DALYs (GBD 2019 Diseases and Injuries Collaborators, 2020). Back and neck pain affect many more people than malaria and tuberculosis, but back and neck pain do not directly cause death. Although the aggregated DALYs are comparable, at the individual level, the health loss of someone suffering from back pain will tend to be much smaller than the health loss suffered by someone with malaria. Assuming that the problems are ‘comparably tractable’, the fair-share principle would recommend that health research into back and neck pain should receive almost equal amounts of funding as malaria and tuberculosis research.

Intuitions about whether this is an appropriate distribution of research resources may diverge. Some, like Joseph Millum, suggest that this would be an inappropriate distribution because more severe causes of health loss—at the individual level—ought to be given extra weight.^7^ In his terminology, the basic proportional view states that, ‘*[i]nsofar as the scientific opportunities are equal*, each patient merits research into their condition proportional to the burden of disease for which that condition is responsible’ (Millum, 2023: 77). Millum then assumes a moderate prioritarianism according to which there are reasons to benefit the worst off—with respect to health—even when doing so does not yield the maximal amount of health benefits overall. This leads him to the severity-weighted proportional view, according to which ‘each person has a claim to health research resources proportional to their *priority-weighted* burden of disease’ (Millum, 2023: 89). By evaluating health research resource allocations against a standard of moderate prioritarianism, Millum captures the important insight that the distribution of harms/benefits matters when it comes to health research priority-setting. This, however, introduces problems all prioritarian views face, namely on what basis to identify the worst off and to decide just how much more weight should be given to them. Note that Millum intends both the basic proportional and severity-weighted views to apply only to ‘disease-specific’ research funding, i.e. that part of a health research budget that has been earmarked for ‘disease-specific’ research.

### Cost-effectiveness and Tractability

Now consider whether the fair-share principle accommodates the idea that investment in health research needs to take cost-effectiveness into account. Roughly, a research programme is more cost-effective than another if it is cheaper to implement to achieve a pre-defined target, or, given a fixed budget, a programme is more cost-effective the larger the benefit it can achieve. Cost-effectiveness therefore incorporates two variables: the economic cost of a research programme and its expected benefit should it succeed. The benefit can be conceptualised in terms of a health-related metric, such as a reduction in DALYs or an increase in life expectancy. Although the fair-share principle includes a clause on ‘tractability’, this is not the same criterion as cost-effectiveness.^8^ Certain projects might be currently intractable from an epistemic and/or material point of view. There might, however, be tractable research programmes, for which epistemic and material resources *are* adequate, but whose implementation would be extremely expensive. This might be the case, for example, for pre-clinical cancer research using radiopharmaceuticals, where the radioactive raw materials are very expensive even though there are clear avenues for further research. Or there might be important, technically feasible public health research to be done on the health effects of urban green spaces, but this multi-methodology, interdisciplinary research may be expensive. Some research may seem so promising, i.e. the health benefits to be gained so large, that the high cost can be justified, but this is a separate consideration from research tractability. Given limited resources, weighing in on whether the (opportunity) cost of research can be justified is an ethical consideration that should not be sidestepped by ‘simply’ applying the fair-share principle. Considerations of tractability and cost-effectiveness thus make the evaluation of actual distributions of health research funding incredibly difficult, as both Kitcher^9^ and Millum^10^ acknowledge.

In sum, the fair-share principle faces three ‘internal’ or general problems: how much priority to give the worst off, how to factor in tractability and how to consider cost-effectiveness. At face value, each of these provides reason for health research resources to be distributed out of proportion with disease burden.

## External Problems: How the Fair-share Principle Sidelines Health Inequalities

In addition to these three general considerations, there is another set of problems for the fair-share principle that arises when the principle is used, as it inevitably has to be, in conjunction with real-world health research budget figures and disease burden estimates. I will argue that the representation of these estimates is value-laden and that the estimates underdetermine health research budget allocation in the sense that even comprehensive estimates are incomplete in a variety of ways. These estimates encourage, and seem to justify, users of the fair-share principle focussing on biomedical research (e.g. Reiss and Kitcher, 2009; Yao *et al.*, 2015; Madsen and Andersen, 2024) while neglecting the complex causal factors that are pivotal to public health.

Recall that to distribute health research resources according to the fair-share principle is to suggest that, waiving considerations of tractability, Disease X, which causes 10 per cent of total suffering from all diseases, ought to receive 10 per cent of the health research budget; Disease Y, which causes 5 per cent of total suffering, ought to receive 5 per cent of the health research budget and so on. This simple allocation mechanism assumes that the collection of all diseases and the total health research budget can be represented as two sets, each containing the same number of elements (each disease is one element in the ‘disease set’ and the budget is subdivided into portions to match the number of diseases) so that there is a one-to-one mapping between the elements of the sets. Does this simple mapping work? In what form is the ‘disease set’ available? What does the health research budget consist of?

### Specifying the Health Research Budget

Starting with the latter question first, recall that in their assessment of malaria and tuberculosis research funding, Flory and Kitcher use estimates for the total global health research budget to conclude that research into these diseases is underfunded when considering global disease burden. Later, they argue that ‘affluent nations … could afford to increase the research budget so that such diseases as cancer were still funded at approximately their current levels … and the entire research budget were constructed by indexing to these amounts by applying the fair share principle’ (Flory and Kitcher, 2004: 63). Gillum *et al.* (2011) and Ballreich *et al.* (2021) scrutinise the NIH budget; the UK Clinical Research Collaboration (2023) evaluates public and charity-funded health research in the UK; Stuckler *et al.* (2008) analyse the WHO budget using the fair-share principle. Any application of the principle needs to specify a budget. This is important because obtaining estimates for health research funding is non-trivial.^11^ Data gaps exist especially for national health research investments in low-income countries, and a detailed breakdown of industry-funded research is often unavailable publicly (cf. Global Forum for Health Research, 2001; Røttingen *et al.*, 2013).^12^ Being clear about which research budget is being invoked in an application of the principle is important both because it clarifies the size of the budget to be apportioned, and it can alert researchers to the need for gathering more data on health research resources.

What is often overlooked by proponents of the fair-share principle and similar proposals, however, is that budgets like that of the NIH and WHO are not designed in purely disease-centric terms. Advocating for total funding to be parcelled out proportionally to disease burden would necessarily reappropriate parts of those budgets that were reserved for other purposes, such as research capacity strengthening and personnel training. What about applying the fair-share principle only to ‘disease-specific’ funding to circumvent this criticism?^13^ This raises the question, given a fixed health research budget, how much of it should be apportioned to disease-specific research versus other types of research and investments (e.g. capacity strengthening and personnel training). Much like the question of how much priority should be given to the worse off, this general question cannot be answered by the principle itself.

Even within ‘disease-specific’ research, however, assigning certain kinds of research to particular diseases will often be difficult. For example, public health research into smoking cessation programmes would be expected to affect multiple diseases (e.g. lung cancer, oesophageal cancer, heart disease etc.)—in what proportion should this research be assigned to these diseases? Similarly, what kind of coordination between research funding allocated to lung cancer and oesophageal cancer and heart disease would be required? This ostensibly practical coordination problem is related to the crux of the matter, namely the value-laden decisions about how to represent or specify the causes of health loss, which I discuss in the following sections.

### Introduction to the Global Burden of Disease Studies

Turning now to the question of which diseases constitute the elements of the ‘disease set’, let’s take a closer look at the GBD studies, given how widely used and easily invoked they are. I will show that the studies’ disease burden estimates reinforce, and seem to justify, users of the fair-share principle focussing on biomedical research, rather than taking into consideration a larger array of health determinants and approaches to health research.^14^

The GBD study was developed in the 1990s (cf. Murray, Lopez and Jamison, 1994) and the most recent study presents estimates for 2023 (GBD 2023 Disease and Injury and Risk Factor Collaborators, 2025). Originally, the study was run by researchers at Harvard University, then moved to the WHO, and is now overseen by the Institute for Health Metrics and Evaluation with funding from the Gates Foundation and Bloomberg Philanthropies (Tichenor and Sridhar, 2020; Eyal *et al.*, 2020a). The overarching aim of these global studies has been to provide a comprehensive estimation of the global burden of diseases, injuries and risk factors, with the derivative aim of using these estimates for policy-making. One of the intended uses of the DALY as an indicator of disease burden was to ‘aid in setting health research priorities’ (Murray, 1994: 429).

The health outcomes of the GBD are grouped at four levels of hierarchical categories, which are ‘mutually exclusive, collectively exhaustive of the mortality and morbidity burden, and relevant to global health policymaking’ (Vos, 2020: 16). Importantly, each health outcome, i.e. each death and each DALY, is *attributed* to exactly one cause derived from the International Classification of Diseases (ICD), which was itself based on a list of causes of death developed in 19th-century France. This so-called categorical attribution derives, historically, from the practice of assigning a single cause of death on death certificates. The ICD then further evolved primarily in clinical settings. Notice how this pressure to choose a single cause collapses a patient’s complex life and medical history into a single code. As a consequence, the ICD has had to adapt over time to include non-fatal and chronic causes of health loss (cf. Bowker and Star, 1999).^15^ Relatedly, due to the ICD’s initial development for clinical purposes, the listed causes are ‘close to the body’ or ‘proximal’ causes of health loss, rather than more ‘distal’ causes.^16^

The use of categorical attribution in the GBD means, first, that the total number of deaths, attributed by cause, will add up to the actual number of total deaths; second, that the numbers cannot be artificially ‘inflated’. For example, if one hundred people die with/from HIV and TB, their deaths are all attributed to HIV, and it would not be possible to say that 100 people died of HIV *and* 100 people died of TB. These features make the resulting estimates intuitive, but the drawback of categorical attribution is that it does not capture the actual causal complexity of health loss (cf. Murray and Schroeder, 2020).

The highest level of the hierarchy, level 1, ‘includes three large cause groupings of NCDs [non-communicable diseases], CMNN [communicable, maternal, neonatal, and nutritional] diseases, and injuries’ (GBD 2023 Disease and Injury and Risk Factor Collaborators, 2025: 1878). Level 2 includes e.g. ‘neoplasms’ and ‘musculoskeletal disorders’ nested under NCDs; ‘maternal and neonatal disorders’ and ‘respiratory infections and tuberculosis’ under CMNN diseases; ‘transport injuries’ and ‘self-harm and interpersonal violence’ nested under injuries. Level 3 becomes more specific: ‘musculoskeletal disorders’ are broken down into ‘low back pain’ and ‘neck pain’ etc.; ‘tuberculosis’ is separated from ‘lower respiratory infections’; ‘self-harm’ is separated from ‘interpersonal violence’. At level 4, there are even more fine-grained distinctions. For example, level 3 ‘gynaecological diseases’ are disaggregated at level 4 into ‘endometriosis’, ‘uterine fibroids’, ‘premenstrual syndrome’, ‘other gynaecological diseases’ etc.^17^ This is indeed a comprehensive attempt at capturing global health loss.

### Global Burden of Disease Estimates Underdetermine Research Priority-Setting

This brief introduction to the GBD will hopefully begin to make clear that stating that e.g. malaria contributes 1.9 per cent of total global DALYs and that a proportional amount of health research funding (e.g. of the total global budget, or from the WHO or NIH) should be invested in malarial research, implies that the remaining 98.1 per cent of that health research budget should (proportionally) be invested in research on the other elements of the ‘disease set’ as the GBD aggregates them, namely, ‘road injuries’, ‘neck pain’, ‘premenstrual syndrome’ etc. However, as this list of diseases and injuries makes clear, there are some elements in that list that are not suitably, or at least not obviously, addressed by research funded by the health research budget. Following the fair-share principle would involve investing 2–3 per cent of the (global) health research budget into research addressing health loss from road injuries, about 1 per cent into research on reducing health loss from interpersonal violence etc. Reducing health loss from some of these causes can and should be achieved through very different means.

What are the grounds of this ‘should’? In what sense is the health research budget unsuited to addressing certain elements in the ‘disease set’? First, there are ‘diseases’ among the GBD estimates that are in some sense intuitively unsuitable to the health research budget, such as road injuries. One way of dealing with this problem when using the GBD estimates is to screen off those causes—such as potentially the entire level 1 injuries category—whose associated health losses are not (or should not be) within the remit of the health research budget. One advantage of this is that the percentage of DALYs lost due to e.g. malaria and tuberculosis would inflate, so one could make an even stronger case for the unjust underfunding of these diseases.^18^ In the case of road injuries, investments into better roads, enforcement of helmet and seat belt regulations, and lower speed limits—funded e.g. by the Department of Transport—may be more appropriate interventions. Unlike much health research funding, embodied by NIH or UK Research and Innovation budgets, investing in safer roads intervenes in the complex causal landscape of health loss at an earlier, preventive stage. Moreover, in the case of avertable road injuries, it would be better, morally speaking, to prevent health loss in the first place than to treat road injuries, even when this is not the most cost-effective option. This is thus an ethical reason for suggesting that the limited health research budget ought not be allocated proportionally to a cause of health loss for which other, more appropriate strategies exist.^19^

Unlike the element ‘road injuries’, there are elements of the GBD ‘disease set’, such as malaria and tuberculosis, about which there is widespread agreement that the health loss they cause could and should be reduced, at least in part, with further insights from health research. In the case of malaria, some research programmes try to better understand the *Plasmodium* parasite with the aim of designing new drugs to interfere with its life cycle. Another approach is to develop more effective vaccines that do not need to be transported and stored at low temperatures. However, even in the case of malaria, other effective approaches may not be biomedical, such as investigating how best to distribute bed nets.

Back and neck pain are examples of elements of the disease set that are plausibly the purview of both health and non-health research budgets. Many cases of back pain are caused by strains sustained while lifting something heavy, and many cases of neck pain are caused by poor posture. One approach to reducing health loss from such pain is by investing in research programmes that aim at developing new or more effective pain medicines.^20^ Another approach—funded by e.g. the Department for Work and Pensions—may look into ‘occupational health’ more generally, for example by designing, testing and implementing new guidelines for ways of working at a desk that reduce the risk of neck injuries.

These examples suggest that most, if not all, elements of the disease set ought to be tackled by some combination of health research and non-health resources. No element in the disease set is plausibly addressed *only* with the resources of a health research budget. Although I will not dwell on this here, the just distribution of health research resources therefore depends on the allocation of other resources in a society and should not be made in isolation. Moreover, this discussion highlights that what we think of as falling within the remit of ‘health research’—even though not everyone will agree exactly about these boundaries^21^—is linked to how we conceive of the causes of health loss. My contention is that the GBD—by representing only ‘proximal’ causes of health loss, especially myriad infectious agents and particular organ pathologies—encourages its users to think in biomedical terms (e.g. Yao *et al.*, 2015; Madsen and Andersen, 2024; Schmallenbach *et al.*, 2025) at the cost of downplaying considerations of a broader public health nature.

### Problems Stemming From the Categorical Attribution of Causes

I now discuss in more detail the implications of the GBD’s use of categorical attribution of causes: assigning a single ‘proximal’ cause to each unit of health loss, thereby simplifying but also distorting the complex causal landscape of actual health losses. We will see that applying the fair-share principle to the ‘disease set’ construed in this way leads to a discrepancy between narrowly defined ‘proximal’ causes, on the one hand, and the variety of ways in which the health losses could be prevented or ameliorated, on the other.^22^

Elaborating on James Woodward’s (2020) and Ned Hall’s (2020) discussions of causal attribution and contribution in the GBD, I outline three important distortions the GBD introduces into the representation of the causal landscape of health loss. First, the practice of categorical attribution—which, to reiterate, arose originally out of the pragmatic requirement to assign a single cause of death to each person in a clinical setting—creates the illusion that each cause listed in the GBD is independent of all other causes.^23^ If each cause were independent, then removing a particular cause would lead to a decrease in health loss/DALYs corresponding *exactly* to the health loss attributed to that cause. However, this independence assumption is rarely met in practice. In some cases, this is because seemingly independent causes of health loss are linked mechanistically. For example, recall that HIV/TB deaths are attributed by convention to HIV; the fair-share principle only ‘sees’ the HIV burden and recommends more research accordingly, which, in this case, may well be an effective way of reducing health loss due to HIV/TB. But does that mean an HIV/TB death lends no support to the idea of investigating more effective antibiotics?^24^ Similarly, DALYs attributed to self-harm in the GBD are linked to a number of mental health disorders, yet the fair-share principle would view these as separate and consequently allocate too little to mental health research.

But even in cases where causes are not linked mechanistically, causes of health loss are often not independent, i.e. they are not additively decomposable. Imagine we could, with the snip of our fingers, eliminate the *Plasmodium* parasites that cause malaria. Would this reduce DALYs in the next iteration of the GBD by exactly the 1.9 per cent currently attributed to malaria? The answer is no because in the next year some of the people who would have suffered from malaria would be affected by other things; some may have a road traffic accident, others may develop diabetes etc. The ‘proximal’ causes listed in the GBD are not independent from one another, and assuming that they are could lead to inefficient or inappropriate research prioritisation.

The second distortion the GBD introduces, which is related to the first, is the representation of these ‘proximal’ causes as together causing 100 per cent of all health loss. This pragmatic decision is justified partly due to its intuitiveness for users of the GBD, including policy-makers (cf. Murray and Schroeder, 2020).

The third distortion is the exclusive focus on ‘proximal’ causes, which in combination with the first two distortions, squeezes out all other, more ‘distal’ causes from the picture, suggesting that they do not contribute to the DALYs. Note that I am not claiming the GBD epidemiologists do not know that more ‘distal’ causes like smoking, or the social determinants of health, contribute to health loss. My claim is that, given the value-laden choice of computing causal attribution estimates from causes that are ‘close to the body’, the GBD studies, mathematically, do not leave room for more ‘distal’ causes.

Given these distortions, what happens when the fair-share principle makes its recommendations based on GBD estimates? First, in drawing on the GBD’s causal representation of health loss, any application of the fair-share principle will obscure the value-ladenness of choices that went into creating this particular representation.

Second, and most importantly, in addition to increasing overall population health, health research priority-setting ought to aim at reducing health inequalities (cf. World Health Organization, 2025). We can see how applying the fair-share principle to GBD estimates risks failing to fulfil this aim: by concentrating on ‘proximal’ causes the principle entrenches a focus on these causes at the expense of more ‘distal’ causes. This is problematic because although the exact mechanisms by which ‘distal’ causes, including the social determinants of health, contribute to health loss are a matter of much research, it is universally accepted that these determinants do contribute to health loss. Importantly, they do so in ways that affect different (sub)populations differentially, with more disadvantaged (sub)populations more adversely affected. The principle fails to be fair because attention to ‘proximal’ causes narrows the focus on, and seems to justify, biomedical interventions, thereby sidelining population and public health research on the social determinants of health and perpetuating health injustices (cf. Goldberg, 2014; Valles, 2021).

Consider, as an example of these issues—the non-independence of causes and the focus on ‘proximal’ causes—attempts to reduce health loss attributed to lung cancer. Reducing health loss from lung cancer could be brought about by a new drug that is highly specific for smoking-induced lung cancer, or by anti-smoking campaigns, or by increasing tax on tobacco, or by some other as-yet untried public health intervention. However, the latter three approaches will also reduce health losses attributed to a number of other cancers as well as smoking-related heart disease. This harks back to the point made earlier about coordinating research funding: there would have to be mechanisms to prevent research funds for lung cancer, oesophageal cancer, heart disease etc. from all only investing in smoking cessation research and from none investing in this type of research. The non-independence of causes is mirrored in the non-independence of interventions/research priorities.

Relatedly, different health research and resultant interventions will have different effects both on the amount of health loss that can be averted and who will benefit most from them. For example, in high-income countries, smoking cessation programmes are more likely to benefit people of higher socioeconomic status (Hiscock *et al.*, 2012), who are also more likely to enrol in clinical trials and thus benefit from new pharmaceuticals (Donzo *et al.*, 2024).^25^ The important point is that when the fair-share principle draws on the GBD estimates and its causal configuration, health research is much more likely to be skewed towards programmes that aim at ‘proximal’ interventions; innovative public health research, which has the potential not only of reducing health loss but also of narrowing health gaps, goes underfunded.

In sum, there is no ‘neutral’ quantitative way of distributing resources ‘in proportion’ with causes of health loss. Any such apportionment has to rely, within certain bounds, on value judgements about which causes to investigate more thoroughly than others. The fair-share principle in conjunction with the GBD ‘disease set’ facilitates this in a non-arbitrary yet value-laden way that focusses on the ‘proximal’ causes at the expense of the social determinants of health.

## Should the Fair-share Principle be Rescued?

Given this extensive critique, should we nonetheless cling to the fair-share principle? The preceding discussion shows in detail why the recommendations of the fair-share principle in conjunction with GBD estimates of disease burden are not an ideal to aim for. However, the principle clearly captures an intuitive starting point for health research resource allocation, namely a (roughly) proportional allocation of resources to *something*. I have argued that that something ought not be the causes of health loss as currently captured by the GBD. In this section, I sketch an alternative proposal that stays true to the fair-share principle’s guiding motivation while addressing health inequities more explicitly.

The general idea is to broaden the non-epistemic values that enter into the representation of causes of health loss by creating parallel versions of the GBD, so that different users can access a version that is suited to their purposes.^26^ To get the fair-share principle off the ground, one pragmatic value—namely the requirement for represented causes of health loss to add up to 100 per cent—has to be taken on board. But in addition to certain pragmatic values and the preference for including only causes that are ‘close to the body’, other values could also be embedded in this causal representation.

Let me illustrate this general idea using two examples. The first example follows directly from the foregoing discussions on ‘proximal’ versus ‘distal’ causes of health loss, and the neglect of public health research. At the moment, GBD researchers invest huge amounts of resources into modelling the DALYs attributable to the 375 causes on their list. One reason this is so effortful is that the attribution has to be modelled—including comorbidity corrections—on the assumption that the causes are independent, which, I argued earlier, is not the case.

I suggest that, at least in theory, it would be possible to include more ‘distal’ causes to this list, such as e.g. smoking and educational attainment. These causes are of course also not independent of one another, i.e. not additively decomposable. In this particular case, educational status likely influences smoking patterns. And, as described above, even when causes are not linked mechanistically, removing a cause is unlikely to yield the exact decrease in health loss attributed to that cause. For example, in a given time frame, although removing smoking as a cause would surely decrease health losses from smoking-related cancers, the people who would have suffered from these may develop other health conditions instead.

While it would certainly not make the modelling easier to include ‘distal’ causes, there is no principled reason why this could not be done. Deliberately incorporating causes that are amenable to public health interventions would boost the proportion of health research resources that go towards public health research when the fair-share principle is applied to that version of the GBD. And this, in turn, would ideally help to reduce health inequalities. Exactly how to model the relative contributions of ‘proximal’ and ‘distal’ causes of health loss, and where to draw the boundary on ‘distal’ causes, are open questions.^27^ Different answers will reflect different values. Importantly, any representation of the causes of health loss will be value-laden, and this needs to be at least acknowledged and at best deliberated upon.

Now consider a second, parallel way in which the GBD could represent causes of health loss. While the inclusion of social determinants of health would improve health research resource allocation in certain regards, this way of carving up the causal landscape may be particularly disadvantageous for sufferers of certain rare diseases. Many genetic rare diseases are heritable, and it is an open empirical question how much they are linked to the social determinants of health, i.e. how much tackling these determinants would reduce DALYs currently attributed to rare diseases. This mirrors a point made above: unlike in the case of road injuries, where there are lots of interventions that do not require health research, some rare diseases may mainly be tractable with more biomedical research. If so, this provides a reason to fund rare disease research beyond its ‘fair share’, as judged by the GBD’s current attribution of causes.^28^

At the moment, the GBD’s approach is to categorise rare diseases based on the primary organ system they affect. For example, Huntington’s disease is lumped under the level 3 category ‘other neurological disorders’; cystic fibrosis under the level 4 category ‘other endocrine, metabolic, blood, and immune disorders excluding thyroid disorders’; sickle cell diseases are their own level 4 category in the ‘other non-communicable diseases’ category. This is one justifiable way to represent these diseases, but there are reasons to make a value-laden decision to lump rare diseases together, e.g. to make a level 2 category called ‘rare diseases’. This would highlight the combined burden these diseases contribute to total DALYs (cf. Chung *et al.*, 2022; Halley *et al.*, 2023), thus potentially boosting health research resource allocation to ‘rare diseases’ when the fair-share principle is applied to this GBD version.^29^ One might object that this would be an ad hoc decision, but this charge could also be levelled at the GBD in its current form: all cancers, regardless of aetiology and affected organ system, are lumped together in the level 2 category ‘neoplasms’. The overarching point is that the GBD’s cause hierarchy is already driven by value-laden decisions; there are no value-free ways of carving up the complex causal landscape of health loss.

## Concluding Remarks

In conclusion, let me reiterate that this proposal is a sketch only, which raises further questions. As alluded to, there are both technical and conceptual questions about how exactly to model a larger number of causes, such as the social determinants of health. Developing these alternative models should happen in conversation with (at least) potential users of the model, which leads to another open question: what would the relationship between different models be and how should users choose which version(s) to use for which purposes?

I am proposing that more and different values—values more closely aligned with the twin aims of public health: improving overall health and reducing health inequalities—ought to be represented in descriptive epidemiological studies like the GBD. Drawing on *these* studies, I suggest, would allow the fair-share principle to advance health justice in a more sustained and transparent manner. Finally, despite various practical hurdles, it is worth remembering that even with this broader conception of the causes of health loss, the distribution of health research resources should not be made independently of the allocation of other, ‘non-health’ resources in society.

## Notes

1 The questions being addressed here are therefore distinct from questions regarding the allocation and rationing of scarce clinical and health *care* resources, such as hospital beds, ventilators and medical staff. These questions receive ample attention in bioethics (e.g. Bognar and Hirose, 2022).

2 There were, of course, parallel debates about reduced provision of non-emergency medical *care* due to the rise of COVID-19-related health care needs.

3 I thank an anonymous reviewer for prompting me to highlight that this is an active and ongoing debate amongst policy-makers.

4 I thank Joseph Millum for pressing me to clarify where I stand on this point.

5 It is a separate question, beyond the scope of this paper, whether DALYs are good measures of ‘total suffering’ due to disease. Although the DALY is a plausible index of health loss, it is a highly value-laden measure. See, for example, Schroeder (2017) and Solberg *et al.* (2020) for critical discussions.

6 Focussing on the allocation of development assistance and health aid rather than health research, Voigt and King (2017) similarly point out that studies evaluating this aid often assume that the aid/assistance should be provided in proportion to disease burden, but that disease burden and need are not necessarily the same thing.

7 I am inclined to agree with Millum on this point, but the ‘external’ problems discussed below persist no matter what view one takes here.

8 By contrast, Millum’s criterion of ‘scientific opportunity’ already captures the notion of cost-effectiveness.

9 ‘Mechanical application of the fair-share principle would be foolish, since considerations about profitable [sic?] inquiry should attend to considerations of research promise. Hence, the formulation given introduced the proviso that issues of relative tractability were waived. Consequently, the actual distribution of research effort might be defended by proposing that the affluent diseases actually investigated—possibly even overstudied—are especially likely to yield important insights.’ (Kitcher, 2011: 122)

10 Any attempt to criticise a particular health research resource distribution ‘face[s] the challenge of distinguishing a justified deviation [from proportionality] from a problematic mismatch’ (Millum, 2023: 90).

11 Specifying the budget is also important because different funders have different responsibilities towards their stakeholders (cf. Pratt and Hyder, 2017; Pierson and Millum, 2018). It would be odd, for example, to charge a charity like Breast Cancer Now with not ‘aligning’ its research funding with overall UK disease burden. What a funder’s responsibilities ought to be—whether a health charity should aim at disease burden alleviation beyond its mission; whether a publicly funded national research body ought to take global rather than national disease burden into account—is an ethical question that the fair-share principle presupposes an answer to.

12 In 2017, the WHO set up a Global Observatory on Health Research and Development with the aim of filling some of these gaps.

13 Recall that Millum’s (2023) severity-weighted proportional view applies only to ‘disease-specific’ funding.

14 Although I focus on the GBD studies here, separate studies carried out to estimate contributions of different causes to health loss, e.g. in Germany (Porst *et al.*, 2022) and Scotland (Grant *et al.*, 2023), face the same issues.

15 For a more recent discussion of the value-ladenness of deciding on ‘the underlying cause of death’ in the context of COVID-19, see Amoretti and Lalumera (2021).

16 For an interesting proposal to drop the proximal/distal terminology, see Krieger (2008).

17 See https://vizhub.healthdata.org/gbd-compare/ for an interactive version of the data.

18 Such an approach has been taken by Madsen and Andersen who compared the disease burden in Denmark—as estimated by the GBD Study—to the Danish research funding landscape. They state, ‘[a]s our focus is on disease research, we exclude the injuries category’ (2024: 5). They go on to show that certain conditions including dysthymia, stroke and alcohol use disorders are ‘underfunded’ compared to the share of total disease burden they cause in Denmark.

19 This approach of screening off categories has a disadvantage. Just because health losses associated with certain elements in the disease set should mainly be addressed by non-health budgets, surely some of the health research budget should go towards research into improved surgical techniques, for example, which may improve health outcomes following unpreventable road injuries. The NIH Fogarty International Center, for example, used to fund such research in low- and middle-income countries through their Global Trauma and Injury Research Training Program.

20 Although his focus is on health law/policy rather than health research priority-setting, Goldberg argues that concentrating on access to pharmacotherapeutics for pain overlooks the ‘long causal pathway between upstream, macrosocial factors and onset of disease/disease outcomes’ (2013: 213).

21 I thank an anonymous reviewer for noting that there will be reasonable disagreement about what ought to be included under the umbrella of ‘health research’.

22 This point echoes early criticisms of those who wanted to use burden of disease measurements to directly (and in some cases proportionally) set health research budget allocations (Williams, 1999; Mooney and Wiseman, 2000). As Ottersen and Norheim put it, ‘[t]he fundamental point was that measures of disease burden focus on the *problem*, while priority setting is—or at least should be—primarily concerned with the *solutions*’ (2020: 294–295).

23 See also Krieger (2017) on this point, although she does not discuss the implications of the non-independence of causes for health research resource allocation.

24 Thanks to Joseph Millum for pointing out that in some iterations of the GBD, HIV/TB is its own category. Would this category license only investments into research that specifically addresses the interplay between these two infections, or each infection on its own but without guidance on relative proportions?

25 Similarly, back pain is not distributed equally within populations; different interventions, from pharmaceutical intervention to better working conditions for manual labourers, will affect different subpopulations differentially, with implications for health-related inequalities (cf. Goldberg, 2013).

26 For further discussion on how to achieve value pluralism in social scientific indicators, see Thoma (2024).

27 For further discussion on the issue of how to draw the boundaries around public health research, see Valles (2018, ch. 4).

28 If we accept a moderate prioritarianism regarding health loss, research into many rare diseases would garner extra weight given many of these diseases’ debilitating nature.

29 How to allocate resources *within* this hypothetical level 2 ‘rare diseases’ category would then still have to be decided.
